# Bioassays Against Pinewood Nematode: Assessment of a Suitable Dilution Agent and Screening for Bioactive Essential Oils

**DOI:** 10.3390/molecules171012312

**Published:** 2012-10-19

**Authors:** Pedro Barbosa, Jorge M. S. Faria, Marta D. Mendes, Luís Silva Dias, Maria Teresa Tinoco, José G. Barroso, Luis G. Pedro, Ana Cristina Figueiredo, Manuel Mota

**Affiliations:** 1 NemaLab-ICAAM, Departamento de Biologia, Universidade de Évora, 7002-554 Évora, Portugal; 2 Universidade de Lisboa, Faculdade de Ciências de Lisboa, DBV, IBB, Centro de Biotecnologia Vegetal, C2, Campo Grande, 1749-016 Lisboa, Portugal; 3 Departamento de Biologia, Universidade de Évora, 7002-554 Évora, Portugal; 4 ICAAM, Departamento de Química, Universidade de Évora, 7000-671 Évora, Portugal

**Keywords:** *Bursaphelenchus xylophilus*, essential oils, nematicide activity, acetone, Triton X-100

## Abstract

Acetone was investigated and found to be an appropriate alternative to Triton X-100 as a solvent of essential oils in bioassays aimed to investigate their effects on pinewood nematode (*Bursaphelenchus xylophilus*) mortality. Therefore it was used as dilution agent to screen the effectiveness of fifty two essential oils against this pest. Thirteen essential oils were highly effective, resulting in more than 90% pinewood nematode mortality at 2 mg/mL, with six of them resulting in 100% mortality. LC_100_ values ranged between 0.50 mg/mL and 0.83 mg/mL for the essential oils of *Origanum vulgare* and *Satureja montana*, respectively. Essential oils were submitted to gas chromatography and gas chromatography-mass spectrometry analysis and their chemical composition established. Data from essential oils with 100% mortality at 2 mg/mL and other essential oils previously found to have LC_100_ ≤ 2 mg/mL was combined, their chemical profiles investigated by correspondences analysis plus automatic classification.

## 1. Introduction

The pinewood nematode (PWN), *Bursaphelenchus xylophilus* L., is a serious threat to forest ecosystems at a global scale, affecting wood trade and its industry [1]. Capable of completely destroying *Pinus* spp. trees, the nematode was classified as an A2 type quarantine pest by the European Plant Protection Organization. In 1999, the nematode was detected for the first time in Portugal, expanding the distribution in the Northern hemisphere (United States, Canada, Mexico, China, Japan, South Korea and Taiwan). More recently, new outbreaks were reported on Madeira Island [2] and in Spain [3]. These developments alerted the EU and new restrictions along with new disease control measures were implemented [4].

Many of the most effective chemicals used for controlling PWN are toxic, expensive or lead to accumulation in the soil, causing significant environmental impacts. In face of the recent EU environmental restrictions, it became necessary to develop environmentally safer control techniques based on natural products. Essential oils have long been known to have significant biological activities. Recent studies have shown that some essential oils appear to have good nematicidal activity against the PWN under laboratory conditions [5,6,7,8].

Triton X-100 is a nonionic detergent-type surfactant, known for its capacity to solubilize membrane proteins. This wetting agent is commonly used for dilution purposes due to the fact it increases the penetrating and spreading properties of liquids. However, our accumulated experience shows that the use of Triton X-100 might be inappropriate for routine use because of the difficulty in obtaining homogenous dissolution of essential oils.

Acetone is a polar aprotic solvent commonly employed for all purpose laboratory uses, particularly given its miscibility with water. Acetone has been rated as a Generally Recognized As Safe (GRAS) substance when present in beverages, baked foods, desserts, and preserves at concentrations ranging from 5 to 8 mg/L [9].

The search for bioactive phytochemicals relies heavily on screening a large number of plant sources followed by bioassay guided fractionation of the most promising ones. Therefore, the present study was set out to: (a) compare the suitability of Triton X-100 and acetone as solvents for plant essential oils to control PWN; (b) widen the screening of species that might be sources of phytochemicals able to completely control PWN.

## 2. Results and Discussion

### 2.1. Assessment of Triton X-100 and Acetone Nematicidal Activity

Mean PWN mortality using Triton X-100 (50 µg/mL) or acetone (1% v:v) was 2.26 ± 0.26% and 1.53 ± 0.19%, respectively, and no significant difference (*p* = 0.027) was found between them. Therefore, and despite the fact that sometimes acetone has been used to control plant parasitic nematodes [10], its use as a solvent for essential oils in bioassays does not raise concerns regarding PWN mortality.

### 2.2. Comparative Evaluation of Essential Oils’ Nematicidal Activity Using Triton X-100 or Acetone as Solvent

Worldwide research in this area employs some kind of detergent (usually Triton X-100) to dilute oils. Plant essential oils have been routinely prepared by serial dilution with distilled water containing Triton X-100 and tested on *B. xylophilus* [6,8,11,12,13,14]. Triton-X is recognized as a good dilution agent for essential oils, able to increase tissue permeability, relatively easy to handle and, because it is not volatile, it allows concentrations to remain essentially constant over time. However, macroscopic examination clearly shows that some essential oils are difficult to dissolve in Triton X-100. One or more oil drops can easily remain inside the detergent and the same can occur when new dilutions are prepared. Ultrasound or temperature increases are commonly used to break up these oils drops. In the present study, when submitting oil/Triton solution to ultrasound irradiation the oil drops remained after 20 min and only started to disappear after 30 min, probably because of a simultaneous temperature increase over time. On the other hand, warming the solution is not feasible given the high volatility of the essential oils. This observation prompted us to check for an alternative solvent. Essential oils of *Cymbopogon citratus*, *Origanum vulgare* and *Satureja montana* previously found to result in more than 90% PWN mortality [8] were tested using acetone as dilution agent and the results compared with those previously found using Triton X-100.

Whenever significant differences were found, the use of Triton X-100 always resulted in reduced PWN mortality compared with acetone. In addition, the variability of effects was always higher using Triton X-100 (Table 1). These results suggest that Triton X-100 may be less effective than acetone in providing a homogeneous solution of essential oils when a dilution series is prepared from a higher concentration, especially in the case of *O. vulgare*. Acetone thus seemed better suited for essential oil dilution in nematicide bioassays, than Triton X-100.

**Table 1:** *Bursaphelenchus xylophilus* mortality (mean ± SE, in percentage) when significant differences were found between the use of Triton X-100 and acetone as dilution agents of essential oils (*p* ≤ 0.006).

Species	Dilution agent	0.25 mg/mL	0.5 mg/mL	1 mg/mL
*Cymbopogon citratus*	Triton X-100 *	14.98 ± 2.17	81.60 ± 1.72	−
	Acetone	83.80 ± 1.08	89.39 ± 1.18	−
*Origanum vulgare*	Triton X-100 *	2.78 ± 0.68	3.72 ± 0.56	26.61 ± 3.83
	Acetone	94.90 ± 1.06	98.81 ± 0.51	100.00 ± 0.00
*Satureja montana* 1	Triton X-100 *	7.13 ± 1.19	−	−
	Acetone	57.60 ± 2.44	−	−

* data from Barbosa *et al.* [8]; − no significant differences between Triton X-100 and acetone treatments (*p* > 0.01).

However, the concentrations tested have no biological meaning *per se* and more important than comparing essential oils effects at defined and more or less arbitrary concentrations is the comparison of biological meaningful parameters derived from the overall response of PWN mortality to a gradient of essential oils concentrations, namely the minimum concentration of essential oil effective against PWN (*l*), the symmetry of the distribution of PWN mortality (*c*) and the minimum concentration of essential oil resulting in 100% PWN mortality (LC_100_).

With *C. citratus* essential oil no significant differences between Triton X-100 and acetone were found in *l* (*p* = 0.090), in *c* (*p* = 0.107), in the maximum mortality (*p* = 0.040) and in the essential oil concentration at which maximum mortality would occur (*p* = 0.015).

Conversely, with *O. vulgare* essential oil, significant differences were found in *l* (*p* = 0.001), *c* (*p* = 2.8 × 10^−8^) and LC_100_ (*p* = 2.7 × 10^−11^). As might be expected from the individual concentration comparisons, LC_100_ values were much higher when Triton X-100 was used (1.984 ± 0.008 mg/mL) than with acetone (0.498 ± 0.028 mg/mL). Previously, using Triton X-100 [8], *O. vulgare* was the only source of essential oil responsible for negative asymmetry in PWN mortality (mean *c* value 4.797 ± 0.109) while all the other essential oils resulted in positive asymmetry. Using acetone, all essential oils resulting in 100% PWN mortality at 2 mg/mL also showed positive asymmetry (Table 2), meaning that interaction of factors occurred, possibly acting multiplicatively [15].

**Table 2:** Estimated values (mean ± SE) of highest concentrations of essential oil at which the mortality is strictly zero (*l*), symmetry of mortality distribution (*c*), and concentrations necessary to result in 100% *Bursaphelenchus xylophilus* mortality (LC_100_).

Species	*l*	*c*		LC_100_
*Origanum vulgare* *	0.100 ± 0.0017 a	1.770 ± 0.095 a	0.498 ± 0.028 a	
*Ruta graveolens* 1	0.096 ± 0.0014 a	1.799 ± 0.052 a	0.571 ± 0.046 b	
*Ruta graveolens* 2	0.095 ± 0.0008 a	1.915 ± 0.135a	0.663 ± 0.032 c	
*Satureja montana* 1 *	0.099 ± 0.0003 a	1.946 ± 0.008 a	0.793 ± 0.002d	
*Satureja montana* 2	0.089 ± 0.0024 b	2.832 ± 0.056 b	0.819 ± 0.007 d	
*Satureja montana* 3	0.089 ± 0.0013 c	2.798 ± 0.020 c	0.828 ± 0.001d	

* Essential oils also tested in Barbosa *et al.* [8] using Triton X-100. Acetone used as solvent. All concentrations in mg/mL. In each column, means with the same letter do not differ for an experiment-wise error rate of 0.01.

**Figure 1:**
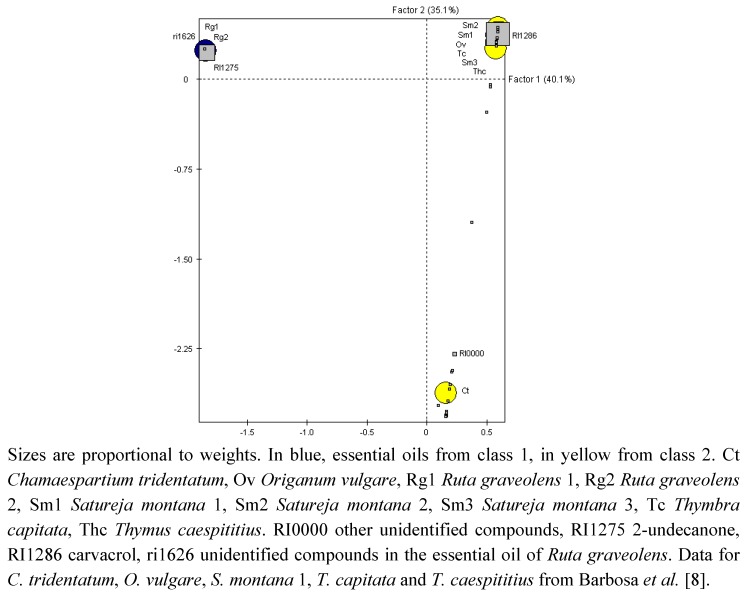
Ordination of essential oils (circles) and constituents (squares) in the first two factors of correspondences analysis.

This discrepancy of *O. vulgare* is surprising and hard to explain given the high similarity between its chemical profile and those of the most part of the other essential oils (Figure 1 and discussion below), but it completely disappears when acetone is used, thereby supporting that using acetone as solvent might be a better choice than using Triton X-100. Finally, with *S. montana* essential oil, no significant differences were found in *l* (*p* = 0.012) and LC_100_ (*p* = 0.352), while c differed significantly (*p* = 0.004) between Triton X-100 (2.310 ± 0.064) and acetone (1.946 ± 0.008), with the latter making more clear the positive asymmetry of PWN mortality distribution.

Macroscopic inspection showed clear solutions without oil drops, revealing that essential oils were completely and homogeneously dissolved. The only downside of this solvent may be its volatility, causing concentration fluctuations in the stock solutions, particularly if kept for long periods. This problem can be diminished if the stock solution is kept at −20 °C until use.

Overall, Triton X-100 replacement by acetone is clearly a sound choice for all practical reasons. Results of PWN response to essential oils using acetone do not differ from results using Triton X-100 or, when they do, acetone based results are more consistent. To our knowledge, despite having been used in the trunk injection technique to control PWN [16], this is the first report on the use of acetone as solvent for essential oil dilution in PWN nematicide bioassays.

### 2.3. Essential Oils Nematicide Activity

Fifty two essential oils isolated from 29 taxa were screened for PWN nematicide activity using acetone as dilution agent (Table 3). As detailed in the next section, for several species, more than one essential oil chemotype, or essential oils isolated from different plant parts of the same species, were assessed.

With 38 of the tested oils, the 99% confidence interval of mean mortality at 2 mg/mL using acetone did not include zero mortality and therefore significant effects of essential oils on PWN occurred (Table 3). However, full mortality was reached with only six oils from three different species, belonging to the Lamiaceae (*O. vulgare* and *S. montana*) and Rutaceae (*Ruta graveolens*).

In general, the response of PWN to different essential oil chemotypes or essential oils isolated from diverse plant parts from the same species varied little or not at all. However PWN mortality in response to *Thymus caespititius* strongly varied with the oils bioassayed, ranging between 6.06 ± 0.62% and 99.44 ± 0.26% but never attaining 100% in all replicates as found previously with an oil from a different population [8].

**Table 3:** Nematicidal activity of 52 essential oils against *Bursaphelenchus xylophilus* exposed for 24 h to a 2 mg/mL solution.

Code	Family / Species	Collection place or source ^b^	Date	Plant part ^c^ Status	I.P. ^d^	Oil yield(%, v/w)	Mortality ^e^ (%)
	**Anacardiaceae**						
*Scm*	*Schinus molle* L.	Évora	2005	Leaves, Fresh	H	0.40	1.54 ± 0.47
	**Apiaceae**						
*Al*	*Angelica lignescens* Reduron et Danton	Flores (Az)	2008	AP (V), Fresh	H	0.08	1.75 ± 0.47
*Cha*	*Chaerophyllum azoricum* Trelease	Flores (Az)	2008	AP (V), Fresh	H	0.25	1.20 ± 0.48
*Fv*1	*Foeniculum vulgare* Miller	Graciosa (Az)	2008	AP (F), Fresh	H	0.33	6.21 ± 0.71 *
*Fv*2	*Foeniculum vulgare* Miller	HS	2008	Seeds, Dried	H	5.61	8.60 ± 0.81 *
*Fv*3	*Foeniculum vulgare* Miller	HS	2008	Seeds, Dried	H	6.09	9.89 ± 1.71 *
*Fv*4	*Foeniculum vulgare* Miller	HS	2008	Seeds, Dried	H	5.88	6.29 ± 0.91 *
*Fv*5	*Foeniculum vulgare* Miller	BPGV	2008	Seeds, Dried	H	4.78	5.95 ± 0.79 *
*Fv*6	*Foeniculum vulgare* Miller	HS	2008	Seeds, Dried	H	1.07	7.13 ± 0.50 *
	**Cupressaceae**						
*Cj*	*Cryptomeria japonica* (L. fil.) D. Don. ^f^	Flores (Az)	2008	Berries, Fresh	H	0.41	0.79 ± 0.39
*Jb*1	*Juniperus brevifolia* (Seub.) Antoine	Flores (Az)	2008	Berries, Fresh	H	0.06	0.84 ± 0.16 *
*Jb*2	*Juniperus brevifolia* (Seub.) Antoine	Flores (Az)	2008	AP (V), Fresh	H	0.45	2.56 ± 0.66
	**Geraniaceae**						
*Pg*1	*Pelargonium graveolens* L’Hér.	Lisboa	2009	AP (V), Fresh	H	0.19	74.79 ± 2.56 *
	**Lamiaceae**						
*Mo*	*Melissa officinalis* L.	HS	2009	AP (F), Fresh	H	0.04	99.30 ± 0.54 *
*Ma*	*Mentha aquatica* L.	HS	2009	AP (F), Dried	H	0.90	7.77 ± 0.83 *
*Mc*1	*Mentha cervina* L.	Beja	2005	AP (F), Fresh	H	2.00	93.56 ± 1.07 *
*Mc2*	*Mentha cervina* L.	HS	2009	AP (V), Dried	H	2.12	92.57 ± 1.48 *
*Ms*	*Mentha spicata* L.	Beja	2009	AP (V), Fresh	H	0.25	47.36 ± 2.22 *
*Nc*	*Nepeta cataria* L.	HS	2009	AP (F), Fresh	H	0.18	22.03 ± 2.66 *
*Ov*	*Origanum vulgare* L. ^a^	Évora	2007	AP (F), Fresh	H	1.70	100.00 ± 0.00 *
*Ro*1	*Rosmarinus officinalis* L.	HS	2009	Leaves, Dried	H	1.95	2.55 ± 0.84
*Ro*2	*Rosmarinus officinalis* L.	Lisboa	2009	AP (V), Fresh	H	0.64	0.40 ± 0.41
*Ro*3	*Rosmarinus officinalis* L.	Lisboa	2009	AP (F), Fresh	H	1.14	2.30 ± 0.52
*So*1	*Salvia officinalis* L.	Lisboa	2009	AP (V), Fresh	H	0.54	1.06 ± 0.40
*So*2	*Salvia officinalis* L.	Lisboa	2009	AP (V), Fresh	H	0.71	0.07 ± 0.43
*Sm*1	*Satureja montana* L.^a^	HS	2008	Leaves, Dried	H	1.60	100.00 ± 0.00 *
*Sm*2	*Satureja montana* L.	HS	2009	AP (V), Dried	H	0.55	100.00 ± 0.00 *
*Sm*3	*Satureja montana* L.	Beja	2009	AP (F), Fresh	D-E	−	100.00 ± 0.00 *
*Tc*1	*Thymus caespititius* Brot.	Madeira	2006	AP (F), Fresh	D-E	−	6.06 ± 0.62 *
*Tc*2	*Thymus caespititius* Brot.	S. Jorge (Az)	2007	AP (F), Fresh	D-E	−	97.01 ± 0.98 *
*Tc*3	*Thymus caespititius* Brot.	Flores (Az)	2008	AP (F), Fresh	H	0.06	94.63 ± 1.30 *
*Tc*4	*Thymus caespititius* Brot.	Corvo (Az)	2008	AP (F), Fresh	H	0.22	99.44 ± 0.26 *
*Tc*5	*Thymus caespititius* Brot.	Gerês	2008	AP (F), Fresh	H	0.35	51.61 ± 3.60 *
*Tc*6	*Thymus caespititius* Brot.	Graciosa (Az)	2008	AP (F), Fresh	H	0.38	58.21 ± 2.19 *
*Tca*	*Thymus camphoratus* Hoffmans. & Link	Faro	2008	AP (F), Fresh	H	0.21	3.30 ± 0.59 *
*Tvl*	*Thymus villosus* ssp. *lusitanicus* (Boiss.) Coutinho	Leiria	2008	AP (F), Fresh	H	1.25	66.85 ± 3.44 *
*Tzs*	*Thymus zygis* ssp. *sylvestris* (Hoffmans. & Link) Coutinho	Leiria	2008	AP (F), Fresh	H	0.23	24.25 ± 3.18 *
	**Lauraceae**						
*Cc*	*Cinnamomum camphora* (L.) T. Nees & C.H. Eberm.	Coimbra	2009	Branches without leaves, Dried	H	0.55	1.56 ± 0.16 *
*La*	*Laurus azorica* (Seub.) J. Franco	Flores (Az)	2008	AP (V), Fresh	H	0.25	2.17 ± 0.66
*Lnc*1	*Laurus novocanariensis* Rivas Mart., Lousã, Fern. Prieto, E. Díaz, J.C. Costa & C. Aguiar	Porto da Cruz, Madeira	2009	Branches, Fresh	H	0.42	2.22 ± 0.39 *
*Lnc*2	*Laurus novocanariensis* Rivas Mart., Lousã, Fern. Prieto, E. Díaz, J.C. Costa & C. Aguiar	Porto da Cruz, Madeira	2009	Branches, Fresh	H	0.48	2.80 ± 0.34 *
*Lnc*3	*Laurus novocanariensis* Rivas Mart., Lousã, Fern. Prieto, E. Díaz, J.C. Costa & C. Aguiar	Ribeiro Frio, Madeira	2009	Branches, Fresh	H	0.39	2.66 ± 0.75
*Lnc*4	*Laurus novocanariensis* Rivas Mart., Lousã, Fern. Prieto, E. Díaz, J.C. Costa & C. Aguiar	Ribeiro Frio, Madeira	2009	Branches, Fresh	H	0.64	2.91 ± 0.44 *
*Lnc*5	*Laurus novocanariensis* Rivas Mart., Lousã, Fern. Prieto, E. Díaz, J.C. Costa & C. Aguiar	S. Vicente, Madeira	2000	Leaves, Fresh	H	0.30	4.46 ± 0.54 *
	**Myrtaceae**						
*Eg*	*Eucalyptus globulus* Labill.	Lisbon	2009	AP (F), Fresh	H	2.15	4.14 ± 0.85 *
	**Pittosporaceae**						
*Pu*1	*Pittosporum undulatum* Vent.	Graciosa (Az)	2008	Berries, Fresh	H	0.21	1.22 ± 0.34
*Pu*2	*Pittosporum undulatum* Vent.	Graciosa (Az)	2008	Leaves, Fresh	H	0.08	1.46 ± 0.44
	**Poaceae**						
*Cyc*	*Cymbopogon citratus* (DC) Stapf. ^a^	Faro	2008	Leaves, Fresh	H	0.80	98.86 ± 0.32 *
	**Rutaceae**						
*Ca*	*Citrus aurantium* L.	Évora	2009	Leaves, Fresh	H	0.31	26.59 ± 1.47 *
*Rg*1	*Ruta graveolens* L.	Évora	2009	AP (V), Fresh	H	2.60	100.00 ± 0.00 *
*Rg*2	*Ruta graveolens* L.	HS	2009	AP (F), Dried	H	0.90	100.00 ± 0.00 *
	**Verbenaceae**						
*Lc*	*Lippia citriodora* Kunth	HS	2009	AP (V), Dried	H	0.19	54.63 ± 3.53 *

Acetone always used as solvent. ^a^ Essential oils also tested in Barbosa *et al.* [8] using Triton X-100; ^b^ Az = Açores; HS = Herbal shop; BPGV = Banco Português de Germoplasma Vegetal; ^c^ AP = aerial part; (V) = in vegetative phase; (F) = in flowering phase; ^d^ I.P. = isolation procedure; isolation was either by hydrodistillation (H) or distillation-extraction (D–E); ^e^ Mortality values with * have 99% confidence intervals not including zero; ^f^ Nowadays included in Cupressaceae, previously Taxodiaceae.

The three term Weibull function [17] could always be fitted to the effects of the six essential oils able to produce 100% PWN mortality at 2 mg/mL. Coefficients of determination ranged between 0.525 and 0.996 (0.868 ± 0.030). Estimated values of Weibull coefficients *l* and *c* and of LC_100_ are summarized in Table 2, together with significant differences among essential oils for an experiment-wise error rate of 0.01.

Essential oils from *S. montana* 2 and 3 were the most active at low dosages given their mean values of *l*. All the remaining essential oils had significantly higher mean values of *l*, but absolute differences were relatively small, implying that the minimum active concentration could not provide the rationale for the choice of essentials oils deserving deeper study. In addition, *l* values intrinsically fail to identify essential oils with the ability to kill 100% of PWN.

By the contrary, LC_100_ values make clear that the essential oil from *O. vulgare* (0.498 ± 0.028 mg/mL) is a promising source for PWN effective control followed by *R. graveolens* 1 (0.571 ± 0.046 mg/mL) and *R. graveolens* 2 (0.663 ± 0.032 mg/mL).

Essential oils for which interactions of effects can be anticipated are better choices for finding one, or at most a few chemicals, able to kill PWN at the lowest possible concentrations [8]. According to this reasoning, essential oils resulting in strongly asymmetric distributions of PWN mortality are preferable. *S. montana* 2 and 3 have *c* values relatively close to 3.25, the lower limit of *c* for a symmetric distribution. Conversely, the remaining essential oils had *c* values indicating a highly positive asymmetry of PWN distribution of mortality, strongly suggesting that multiplicative interactions of effects occurred.

In short, essential oil from *O. vulgare* closely followed by those from *R. graveolens* 1 and 2 appear as promising sources of phytochemicals worth being selected for bioassay-guided search of highly active compounds able to provide an effective control of PWN.

Variability in essential oil composition and yield is known to occur, particularly due to physiological variation, environmental conditions, and geographic variation [18]. Despite differences due to tested concentration and/or plant part employed to obtain the oil, our results for oils with low effect are similar to those previously obtained for *Cinnamomum camphora* [19], *Citrus aurantium* [11], *Eucalyptus globulus* [11,19], *Lippia citriodora* [20] and *Rosmarinus officinalis* [11].

Also, results for effective oils match those previously obtained with *C. citratus* [11,19], results for *O. vulgare* are different from those previously obtained by Kong *et al.* [11]. Differences in the composition of the essential oils used may explain the different results obtained.

To our knowledge this is the first report of nematicide activity against PWN by *R. graveolens*. Among several properties, essential oils from *O. vulgare*, *R. graveolens* and *S. montana* showed antibacterial [21,22,23] and antifungal [24,25] capability.

### 2.4. Chemical Profile of Essential Oils

Of the 52 essential oils isolated and chemically characterized, only those of *S. montana* (*Sm*2 and *Sm*3) and *R. graveolens* (*Rg*1 and *Rg*2) are detailed in Table 4, since they were the only ones that revealed 100% nematicide activity. Data for *O. vulgare* and *Sm*1 can be found elsewhere [8].

**Table 4:** Chemical composition of essential oils and volatiles of Portuguese plants resulting in 100% mortality of *Bursaphelenchus xylophilus* at 2 mg/mL.

		Lamiaceae		Rutaceae	
Compounds	RI ^a^	*Sm*2	*Sm*3	*Rg*1	*Rg*2
2-Methyloctane	887	−	−	t ^b^	t
Tricyclene	921	t	t	−	−
α-Thujene	924	0.3	2.4	−	−
α-Pinene	930	1.6	2.3	−	−
Camphene	938	1.6	0.1	−	−
1-Octen-3-ol	961	t	t	−	−
β-Pinene	963	0.2	1.2	−	−
*n*-Octanal	973	−	−	t	t
β-Myrcene	975	t	2.7	−	−
α-Phellandrene	995	t	0.4	−	−
δ-3-Carene	1000	t	0.1	−	−
α-Terpinene	1002	0.3	4.1	−	−
*p*-Cymene	1003	20.3	8.1	−	−
1,8-Cineole	1005	t	t	−	−
β-Phellandrene	1005	t	0.1	−	−
Limonene	1009	0.6	0.5	−	−
*cis*-β-Ocimene	1017	t	t	−	−
γ-Terpinene	1035	4.3	41.1	−	−
*trans*-Sabinene hydrate	1037	t	t	−	−
2-Nonanone	1058	−	−	t	t
2,5-Dimethyl styrene	1059	t	t	−	−
Terpinolene	1064	0.4	t	−	−
*cis*-Sabinene hydrate	1066	t	t	−	−
*n*-Nonanal	1073	−	−	t	t
Linalool	1074	t	t	−	−
Geigerene isomer	1116	−	−	t	t
Geigerene	1121	−	−	0.5	0.1
Borneol	1134	3.9	0.1	−	−
Terpinen-4-ol	1148	2.3	0.2	−	−
α-Terpineol	1159	t	t	−	−
2-Decanone	1166	−	−	t	t
Carvacrol methyl ether	1224	3.7	t	−	−
2-Undecanone	1275	−	−	94.4	92.8
Thymol	1275	15.2	t	−	−
Carvacrol	1286	40	35.3	−	−
β-Bourbonene	1379	t	t	−	−
2-Dodecanone ^c^	1389	−	−	t	t
β-Caryophyllene	1414	2.6	1.1	−	−
β-Copaene	1426	t	t	−	−
Aromadendrene	1428	0.3	t	−	−
α-Humulene	1447	t	t	−	−
2-Tridecanone	1479	−	−	t	t
β-Bisabolene	1500	0.1	t	−	−
*trans*-Calamenene	1505	0.1	t	−	−
δ-Cadinene	1505	0.1	0.1	−	−
β-Caryophyllene oxide	1561	0.6	t	−	−
UI *Rg* ^d^	1626	−	−	5.1	7.1
% of identification		98.6	99.9	94.9	92.9
Grouped components					
Monoterpene hydrocarbons		29.6	63.1	−	−
Oxygen-containing monoterpenes		65.2	35.6	−	−
Sesquiterpene hydrocarbons		3.2	1.2	−	−
Oxygen-containing sesquiterpenes		0.6	−	−	−
C13 compounds		−	−	0.5	0.1
Others		−	−	94.4	92.8

*Satureja montana* 2 (*Sm*2), *S. montana* 3 (*Sm*3), *Ruta graveolens* 1 (*Rg*1), *R. graveolens* 2 (*Rg*2). ^a^ RI = Retention index relative to C_8_-C_17_ *n*-alkanes on the DB1 column; ^b^ t = trace (<0.05%); ^c^ identification based on mass spectra only; ^d^ unidentified compound in *R. graveolens* essential oil; *m/z* (rel. int.) 186 [M]^+^ (3), 105 (12), 104 (62), 92 (18), 91 (68), 82 (12), 71 (37), 65 (17), 58 (17), 43 (100).

*O*. *vulgare* (Lamiaceae) essential oil was dominated [8] by carvacrol (36%), γ-terpinene (24%) and *p*-cymene (14%). Carvacrol was also a major component (35%–40%) of the essential oils of the three *S. montana* samples. Differences between essential oils of samples *Sm*1 [8] and *Sm*3 were quite few and their major constituents, by descending order, were γ-terpinene (40%–41%), carvacrol and *p*-cymene (7%–8%). However, differences were more considerable relative to sample *Sm*2, where *p*-cymene (20%) was present in higher concentration and thymol (15%) replaces γ-terpinene in the top three constituents.

Essential oils from fresh (1) and dried (2) *R*. *graveolens* (Rutaceae) were quite similar and characterized by few compounds. Only two compounds displayed major differences: 2-undecanone was more abundant in the fresh form, while an unidentified compound (UI *Rg*, Table 4) was more abundant in the dry form.

The volatile profile of the two Lamiaceae species reported as having nematicide activity was in accordance with previous studies on *O*. *vulgare* [26,27] and *S*. *montana* [27,28]. For *R. graveolens*, previous studies also show 2-undecanone as the main component of the essential oil, although attaining only 34%–47% [29,30].

Chemical profiles of essential oils may provide useful guidelines to design efficient strategies to identify chemicals to be used for PWN control. In fact, essential oils of *R. graveolens* are almost completely constituted by 2-undecanone (94.4% and 92.8% respectively), a compound absent from the essential oils not only of *S. montana* but also from the essential oils previously found to completely control PWN except in the case of *Chamaespartium tridentatum* were it was found in a relatively small amount [8]. Correspondences analysis of the percentage composition of the essential oils, which showed effective nematicide activity both in the present and in a previous study [8], supported the chemical differences between *R. graveolens* essential oils and all other tested essential oils (Figure 1).

The multidimensional pattern of chemicals composition strongly relies upon the amount of 2-undecanone and carvacrol, separating *R. graveolens* essential oils from the remaining oils by the amounts of those compounds. Hierarchical classification performed on the first two factors identifies two classes. One composed by *R. graveolens* essential oils and characterized by significantly high amounts of 2-undecanone and of an unidentified compound of *R. graveolens*, the other composed by all other essential oils and characterized by significantly low amounts of the same compounds.

Altogether these results strongly suggest that 2-undecanone could be the responsible for the effects of *R. graveolens* and simultaneously that one or more compounds not present in *R. graveolens* are also capable of completely control PWN.

Considering only the major constituents of essential oils other than those obtained from *R. graveolens*, LC_50_ in PWN treated with thymol and carvacrol was found to be 1.08 mg/mL and 1.23 mg/mL and higher than 20 mg/mL with *p*-cymene and γ-terpinene [31]. However, LC_100_ associated with *R. graveolens* essential oils (0.617 ± 0.030 mg/mL) is significantly lower (*p* = 3.5 × 10^−^^9^) than LC_100_ associated with all the remaining essential oils (1.156 ± 0.072 mg/mL) and well below concentrations responsible for LC_50_ of single compounds. It is also well below concentrations responsible for LC_50_ of 2-undecanone applied alone to two species of root knot nematodes [32]. Therefore, 2-undecanone, the most likely responsible for the effects found with *R. graveolens*, is clearly a highly promising candidate for PWN control. To our knowledge, this is the first report of the nematicidal activity of this compound against PWN.

## 3. Experimental

### 3.1. Plant Material

The aerial parts of several Portuguese flora species, from collective or individual samples, were collected from wild-growing plants in mainland Portugal and in the Madeira and Açores islands (Portugal). Plant material was stored at −20 °C until extraction. Dried aerial parts from commercially available products sold in local herbal shops were also evaluated. In total, 52 essential oils from 29 taxa representing 12 families (Table 3) were evaluated for nematicide activity.

### 3.2. Essential Oils and Volatiles Extraction

Essential oils were isolated by hydrodistillation (H) for 3 h using a Clevenger-type apparatus according to the European Pharmacopoeia method [33]. Volatiles were isolated by distillation-extraction (D–E) for 3 h using a Likens-Nickerson-type apparatus with 50 mL of distilled *n*-pentane (Riedel-de Haën, Sigma-Aldrich Laborchemikalien GmbH, Seelze, Germany) as the organic solvent (Table 3). Both isolation procedures were run at a distillation rate of 3 mL/min and, on average, at least 100 g of each plant was extracted. The D–E oils recovered in distilled *n*-pentane were concentrated at room temperature under reduced pressure on a rotary evaporator, collected in a vial, and concentrated to a minimum volume, again at room temperature, under nitrogen flux. Essential oils and volatiles were stored in the dark at −20 °C until analysis.

### 3.3. Rearing and Collection of Nematodes

Wood chips from a maritime pine (*Pinus pinaster* Ainton) tree displaying wilt symptoms were collected in the Setúbal region, Portugal. Collected PWN were maintained in Petri dishes containing *Botrytis cinerea* cultured on malt extract agar. Prior to testing, cultured nematodes were separated from the agar medium for 48 h in a Baermann tray [34], placed in a new fungal mat, and left to multiply for one week at 25 °C in the dark. Nematodes were separated from the culture medium as described above and counted under a binocular microscope Olympus SZX-12 (Olympus Corporation, Tokyo, Japan). A nematode suspension in distilled water was made by a series of dilutions, such that 100 µL contained between 100 and 200 mixed-stage nematodes. The suspension was prepared immediately prior to use. The same PWN isolate was employed in a previous study [8].

### 3.4. Bioassays

Bioassays were performed in 96-well microtiter plates (Carl Roth GmbH + Co. KG, Karlsruhe, Germany). In each well, the nematode suspension (99 µL) was added, followed by the essential oil solution (1 µL) diluted in the assessed solvent. Plates were placed in a vortex apparatus at 500 rpm for 2 min and stored at 25 °C in the dark. After 24 h, dead and live nematodes were counted under a binocular microscope (Olympus SZX-12). Nematodes were considered dead if they did not move even when mechanically stimulated.

Comparison of Triton X-100 (50 µg/mL) and acetone (1% v:v) effects on PWN mortality was done preparing 31 wells with the nematode suspension (99 µL), followed by 1 µL of Triton X-100 (Scharlau Chemie, Barcelona, Spain) in distilled water solution (5 g/mL) or 1 µL of acetone (Carl Roth GmbH + Co. KG, Karlsruhe, Germany; 99.8% purity) instead of Triton X-100; PWN mortality was recorded.

The same essential oils of *C*. *citratus*, *O*. *vulgare* and *S*. *montana* previously found [8] to result in more than 90% PWN mortality, at 2 mg/mL in Triton X-100 were used to compare essential oils effects on PWN mortality using Triton X-100 and acetone by testing oils at 2, 1, 0.5, 0.25 and 0.125 mg/mL using acetone as diluent. The higher concentration was prepared from the pure oil and acetone (99.8% purity) which was included as control. The following dilutions series were prepared from the initial one. Each concentration was prepared once and five wells were used per essential oil and concentration. Mortality was recorded as described above and results compared with those previously found using Triton X-100.

Forty nine additional essential oils were tested at 2 mg/mL using acetone as dilution agent. Concentrations were prepared once and five wells were used per essential oil and concentration. Essential oils resulting in 100% mortality at 2 mg/mL in the five wells were further tested at 1, 0.5, 0.25 and 0.125 mg/mL as described above.

### 3.5. Determination of Essential Oils Composition

#### 3.5.1. Gas Chromatography (GC)

Gas chromatographic analyses were performed using a Perkin Elmer Autosystem XL gas chromatograph (Perkin Elmer, Shelton, CT, USA) equipped with two flame ionization detectors (FIDs), a data handling system, and a vaporizing injector port into which two columns of different polarities were installed: a DB-1 fused-silica column (30 m × 0.25 mm i.d., film thickness 0.25 μm; J & W Scientific Inc., Rancho Cordova, CA, USA) and a DB-17HT fused-silica column (30 m × 0.25 mm i.d., film thickness 0.15 μm; J & W Scientific Inc.). Oven temperature was programmed to increase from 45 to 175 °C, in 3 °C/min increments, then up to 300 °C in 15 °C/min increments, and finally held isothermal for 10 min. Gas chromatographic settings were as follows: injector and detectors temperatures, 280 °C and 300 °C, respectively; carrier gas, hydrogen, adjusted to a linear velocity of 30 cm/s. The samples were injected using a split sampling technique, ratio 1:50. The volume of injection was 0.1 μL of a pentane-oil solution (1:1). The percentage composition of the oils was computed by the normalization method from the GC peak areas, calculated as a mean value of two injections from each oil, without response factors.

#### 3.5.2. Gas Chromatography-Mass Spectrometry (GC-MS)

The GC-MS unit consisted of a Perkin Elmer Autosystem XL gas chromatograph, equipped with DB-1 fused-silica column (30 m × 0.25 mm i.d., film thickness 0.25 μm; J & W Scientific, Inc.) interfaced with Perkin-Elmer Turbomass mass spectrometer (software version 4.1, Perkin Elmer). GC-MS settings were as follows: injector and oven temperatures were as above; transfer line temperature, 280 °C; ion source temperature, 220 °C; carrier gas, helium, adjusted to a linear velocity of 30 cm/s; split ratio, 1:40; ionization energy, 70 eV; scan range, 40–300 u; scan time, 1 s. The identity of the components was assigned by comparison of their retention indices relative to C_8_-C_17_ *n* alkane indices, and GC-MS spectra from a laboratory made library based upon the analyses of reference oils, laboratory-synthesized components, and commercial available standards.

### 3.6. Data Analysis

Effects of Triton X-100 and acetone on PWN mortality were compared by exact two-tailed Student *t* test after checking for homocedasticity using the two-tailed *F* distribution.

To account for the observed mortality in controls (M_0_), mortality in treatments (M_T_) was corrected by the Schneider-Orelli’s formula M_C_ = M_T_ − M_0_ / 100 − M_0_ [35] and expressed as percentage. Confidence intervals of 99% for M_C_ were used to identify essential oils active against PWN. M_C_ values at the tested essential oil concentrations using acetone were compared to M_C_ values recorded at the same concentrations using Triton X-100 by exact or approximate two-tailed Student *t* tests after checking for homocedasticity using the two-tailed *F* distribution.

The relation between M_C_ and essential oil concentration was investigated by fitting the Weibull function [17] by least squares nonlinear regression without replication using the Marquardt method [36].

The three parameter Weibull function is expressed as M_C_ = 1 − exp − {[(X − *l*)/*k*]*^c^*} where M_C_ is the observed corrected mortality (in proportion) at essential oil concentration X. *l* is a location parameter that for all practical purposes represents the minimum concentration of essential oil effective against PWN. *k* is a scale parameter that represents the concentration at which the mortality is approximately 63% (LC_63_) when *l* = 0. To control PWN effectively LC_63_ is clearly a less than desirable target and values of LC_100_ were calculated from fitted equations, since in the first situation the remaining population is able to quickly multiply and achieve the previous abundance. *c* is a shape parameter that evaluates the symmetry of the distribution with 3.25 ≤ *c* ≤ 3.61 showing symmetry and representing a good approximation to the normal distribution, *c* < 3.25 positive, *c* > 3.61 negative asymmetry [37,38].

Replicates were defined by their rank of corrected mortality and fitted equations were only accepted after a consistency check of parameter estimates and mortality predictions against original data. *l*, *c* and LC_100_ values using acetone were compared to *l*, *c* and LC_100_ values using Triton X-100 by exact or approximate two-tailed Student *t* tests after checking for homocedasticity using the two-tailed *F* distribution. The effects of essential oils on *l*, *c* and LC_100_ were compared using a least squares linear regression approach with dummy variables to prevent the occurrence of lack of “transitivity” [39,40]. Forward stepwise selection with replication was used and the candidate model included qualitative variables only, namely the species source of the essential oil (coded as 1, 0), with an experiment-wise type I error rate of 0.01 for coefficients calculated using Dunn-Šidák method [41,42]. A significant level of *p* = 0.01 was used throughout. Results of bioassays are presented as means ± SE.

Essential oils chemical profiles resulting in 100% PWN mortality at 2 mg/mL tested with acetone, Triton X-100 or both (Table 2 and [8]) were investigated by correspondences analysis followed by hierarchical classification using the generalized Ward criterion [43]. Chemicals occurring as trace were set as 0.01%. Characterization of factors in correspondences analysis was done using absolute contributions. Classes were characterized by test values of variables with an experiment-wise type I error rate of 0.01 using Dunn-Šidák method [41,42]. LC_100_ values for classes were compared by one-tailed approximate *t* test after checking for homocedasticity using the two-tailed *F* distribution.

## 4. Conclusions

Acetone seems better suited for essential oil dilution in nematicidal bioassays than the commonly employed Triton X-100. To our knowledge this is the first report on the use of acetone as a way to dissolve essential oils in this kind of research. Essential oils from *O. vulgare* closely followed by those from *R. graveolens* appear as promising sources of phytochemicals worth being selected for bioassay-guided search. This is also the first report of nematicide activity against PWN by *R. graveolens*.

In our opinion LC_100_ is preferable to LC_50_ as a way to assess the number of plants with nematicidal activity and strengthen the obtained results.
